# Associations of subjective and objective cognitive functioning after COVID-19: A six-month follow-up of ICU, ward, and home-isolated patients

**DOI:** 10.1016/j.bbih.2023.100587

**Published:** 2023-01-05

**Authors:** Riikka E. Pihlaja, Lina-Lotta S. Kauhanen, Henriikka S. Ollila, Annamari S. Tuulio-Henriksson, Sanna K. Koskinen, Marjaana Tiainen, Viljami R. Salmela, Johanna Hästbacka, Laura S. Hokkanen

**Affiliations:** a Department of Psychology and Logopaedics, Faculty of Medicine, University of Helsinki, Helsinki, Finland; b Department of Anaesthesiology, Intensive Care and Pain Medicine, Helsinki University Hospital and University of Helsinki, Helsinki, Finland; c Division of Neuropsychology, HUS Neurocenter, Helsinki University Hospital and University of Helsinki, Helsinki, Finland; d Department of Neurology, Helsinki University Hospital and University of Helsinki, Helsinki, Finland

**Keywords:** COVID-19, Subjective cognitive symptoms, Cognitive dysfunction

## Abstract

**Background:**

Subjective and objective cognitive dysfunction are reported after COVID-19 but with limited data on their congruence and associations with the severity of the acute disease. The aim of this cohort study is to describe the prevalence of subjective and objective cognitive dysfunction at three and six months after COVID-19 and the associations of subjective cognitive symptoms and psychological and disease-related factors.

**Methods:**

We assessed a cohort of 184 patients at three and six months after COVID-19: 82 patients admitted to the Intensive Care Unit (ICU), 53 admitted to regular hospital wards, and 49 isolated at home. A non-COVID control group of 53 individuals was included. Demographic and clinical data were collected. Subjective cognitive symptoms, objective cognitive impairment, and depressive and post-traumatic stress disorder (PTSD) symptoms were assessed.

**Results:**

At six months, subjective cognitive impairment was reported by 32.3% of ICU-treated, 37.3% of ward-treated, and 33.3% of home-isolated patients and objective cognitive impairment was observed in 36.1% of ICU-treated, 34.7% of ward-treated, and 8.9% of home-isolated patients. Subjective cognitive symptoms were associated with depressive and PTSD symptoms and female sex, but not with objective cognitive assessment or hospital metrics.

**Conclusions:**

One-third of COVID-19 patients, regardless of the acute disease severity, reported high levels of subjective cognitive dysfunction which was not associated with results from objective cognitive screening but with psychological and demographic factors. Our study stresses the importance of thorough assessment of patients reporting long-term subjective symptoms, screening for underlying mental health related factors such as PTSD or depression.

## 1 Introduction

COVID-19 can have sequelae lasting for months after initial recovery (Lopez-Leon et al., 2021), including neurological (Chou et al., 2021) and psychiatric (Premraj et al., 2022) manifestations and cognitive impairment (Daroische et al., 2021). Cognitive impairment is one of the most common prolonged symptoms of COVID-19, as defined by the WHO consensus on post COVID-19 condition (WHO, 2021; Davis et al., 2021). The incidence of cognitive symptoms has ranged from 0 to 78% (Schou et al., 2021) in studies of the acute and post-acute phases of COVID-19 (Krishnan et al., 2022; Zhou et al., 2020). The heterogeneity of study samples and methods may partly explain the large variability in the results. Moreover, few studies have included a control group (Tavares-Junior et al., 2022), which would facilitate differentiating direct effects of the disease and indirect factors of the pandemic (Schou et al., 2021).

There is still limited information on the long-term cognitive outcome of patients with different severity levels of the acute disease (Hampshire et al., 2021), and particularly in mild COVID-19 (Schou et al., 2021; Van Kessel et al., 2022; Vanderlind et al., 2021). Patients who have been treated in the intensive care unit (ICU) have been found to have more cognitive impairment compared to other hospitalized patients (Taquet et al., 2021), although no differences between these patient groups have been found in a three-month follow-up (Rass et al., 2022). Compared to home-treated patients, hospitalization may be a risk to having more cognitive impairments (Becker et al., 2021). However, persisting cognitive symptoms and mental distress may appear also among patients with mild COVID-19 (Van Kessel et al., 2022).

Cognitive functioning can be evaluated using objective assessment or subjective self-reporting questionnaires. COVID-19 patients have commonly reported subjective cognitive complaints (Vanderlind et al., 2021) with as much as 54.7% reported in a 1-year follow-up (Ferrucci et al., 2022). However, there are contradictory results on how self-reported cognitive issues reflect objective cognitive evaluation (Almeria et al., 2020; Blackmon et al., 2022; Miskowiak et al., 2021). Besides infection-related factors, other mechanisms such as education, age, and psychological factors including distress may modulate cognitive outcomes (Majoka and Schimming, 2021). COVID-19 survivors may experience mental distress, including symptoms of depression (Renaud-Charest et al., 2021) and post-traumatic stress disorder (PTSD) (Heesakkers et al., 2022). Because of the symptomatic heterogeneity of COVID-19, studies employing a holistic approach combining clinical assessments with patient-reported measures and medical data are called for (Wahlgren et al., 2022). Additionally, longitudinal assessment across recovery is needed to assess the consequences of COVID-19 over time (Vanderlind et al., 2021).

Our aim was to assess subjective and objective cognitive dysfunction up to six months after COVID-19 in three different levels of acute disease severity: patients treated in the ICU or regular hospital wards or isolated at home. We examined the prevalence of subjective and objective cognitive impairment and the associations of subjective cognitive impairment to objective cognition, symptoms of depression and PTSD, and demographic and disease-related factors at three and six months after COVID-19.

## 2 Materials and methods

### 2.1 Study design, setting, and population

This is a post hoc study of the RECOVID project, a multidisciplinary, single-center, prospective cohort study. The study protocol has been previously described (Ollila et al., 2022). The study is registered at ClinicalTrials.gov (NCT04864938). The Ethics Committee of Helsinki University Hospital (HUS-1949-2020) approved the protocol. All participants gave written informed consent to the study and the principles of the Declaration of Helsinki were followed.

**Fig. 1:**
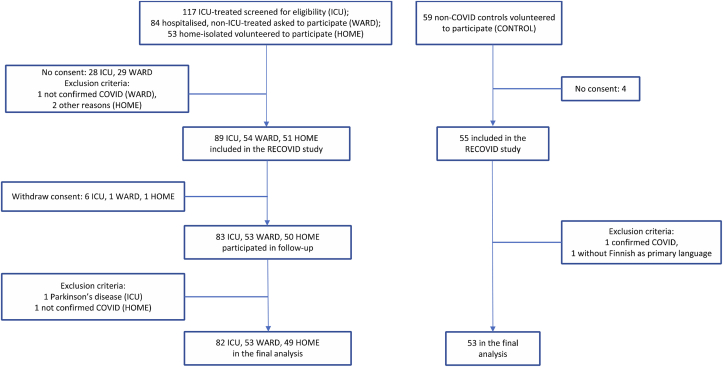
Flow chart of the data compilation.

We assessed COVID-19 patients in three groups of acute disease severity (Fig. 1). A control group who reported no history of COVID-19 was also recruited (CONTROL group). The assessments were conducted three and six months after hospital discharge (ICU and WARD groups) or positive PCR test (HOME group). We included patients with data from at least one cognitive measure in at least one time point, resulting in a final study sample of 184 patients: 82 treated in ICU, 53 hospitalized who received ward-level care (WARD), and 49 home-isolated patients (HOME). The patients had been hospitalized or tested positive for COVID-19 between March 13 and December 31, 2020. Patients were typically admitted to the ICU if they had respiratory rate 30 and oxygen saturation with pulse oximeter 92% or below, were rapidly deteriorating with obviously increased work of breathing, or had other organ dysfunction necessitating intensive support or monitoring. The ICU admission criteria were in broad accordance with the published criteria for severe critical COVID-19 (Alhazzani et al., 2021). Recruitment took place by mailed invitation after discharge (ICU), from a pulmonary outpatient clinic appointment or during acute care in a pulmonology ward (WARD), or by media announcements (HOME and CONTROL). Inclusion criteria included a positive acute phase PCR or antibody test, age 18 or older, and Finnish as primary language. Exclusion criteria were pregnancy, prior major neurological diagnosis, developmental intellectual disability, or severely impaired hearing or vision.

### 2.2 Study outcomes

The main outcomes were the prevalence of subjective and objective cognitive impairment at 3 and 6 months after COVID-19 in three groups of acute disease severity compared to non-COVID controls. Secondary outcomes were the associations of subjective cognitive symptoms with objective cognitive impairment, demographic and disease-related factors, and depressive and PTSD symptoms.

We evaluated subjective cognitive impairment with the A-B Neuropsychological Assessment Schedule (ABNAS) questionnaire (Aldenkamp et al., 1995), a scale of patient-perceived cognitive functioning. It includes 24 items regarding cognitive impairments in daily life. Each item is scored on a scale from 0 to 3 with 0 indicating no difficulty, 1 mild difficulty, 2 moderate difficulty, and 3 severe difficulty. Cognitive symptoms are evaluated on six domains: fatigue (5 questions), slowing (5), memory (4), concentration (4), motor coordination (3), and language (3). The total score ranges from 0 to 72 with higher scores reflecting greater levels of subjective impairment. Aldenkamp et al. (2002) have proposed a dichotomization into low (≤15) and high (>15) scores reflecting low and high levels of subjective cognitive impairment.

We screened objective cognitive functioning with Montreal Cognitive Assessment (MoCA). The telephone-based version (t-MoCA) (Katz et al., 2021) was used at 3 months. It includes a subset of items from the full MoCA, excluding items requiring visual stimuli and pencil-and-paper drawing. It consists of 22 items divided into domains of memory (5 points), attention (6 points), language (3 points), verbal reasoning (2 points), and orientation (6 points). In the 6-month assessment, we used the full version of MoCA, which has additional visuospatial/executive (5 points) and naming (3 points) domains. Total scores consist of points earned plus an additional point given with ≤12 years of education (no extra point is given with a perfect score) resulting in maximal total scores of 22 for t-MoCA and 30 for MoCA. Impaired MoCA scores were defined as ≤19 for t-MoCA (Pendlebury et al., 2013) and ≤25 for MoCA (Nasreddine et al., 2005). Experienced neuropsychologists or clinician investigators trained by experienced neuropsychologists performed the assessments.

We assessed post-traumatic stress disorder (PTSD) symptoms with the Impact of Event Scale-6 (IES-6), an abbreviated 6-item version of the IES-Revised (IES-R) that uses a five-point Likert scale ranging from 0 to 4 (Hosey et al., 2019). The score is formed as the mean of six items and the cut-off point indicating significant clinical symptoms of PTSD has been defined as 1.75 (Hosey et al., 2019).

We evaluated depressive symptoms with the two-question screening method (Depression screen) (Arroll et al., 2003; Whooley et al., 1997) at three months and the Patient Health Questionnaire-9 (PHQ-9) (Kroenke et al., 2001) at six months. The Depression screen was considered positive when “yes” was given to either of the two questions (Arroll et al., 2003). For the PHQ-9, a score of 10 or higher reflects at least moderate depressive symptoms (Kroenke et al., 2001).

We retrieved demographic information and clinical variables (length of hospital and ICU stay, supplementary oxygen, delirium, invasive mechanical ventilation [IMV], Charlson comorbidity index [CCI], and comorbidities) from medical records. Education level was reported by the patient.

### 2.3 Statistical analysis

We provide descriptive statistics for the demographic and clinical characteristics of each group and report the total sample size available for each data element. Sample size was based on power calculations for finding group differences which indicated that n > 50 would be required for each group. Continuous variables are presented as mean (SD) or median (interquartile range [IQR]) and categorical variables as *n* (percentage). Group differences were assessed with ANOVA for normally distributed continuous variables, Kruskal-Wallis for non-normally distributed variables, and χ^2^ test for categorical variables. Changes in the frequency of impairment in follow-up were assessed with McNemar tests. Because of the differences in age and education levels in the groups, we used additional age- and education-adjusted ANCOVAs with Tukey's post hoc tests to assess differences in MoCA scores. We transformed all ABNAS subscale scores to a range of 0–3 to enable comparison of subscales with differing numbers of items and assessed group differences with ANOVA.

We assessed the correlations of subjective cognitive symptoms with depressive and PTSD symptoms, demographic factors, hospital metrics, and objective cognitive impairment with Pearson's or Spearman's r correlations. We then used partial Pearson correlation coefficients controlling for age, education, and IES-6 to assess adjusted associations between subjective cognitive symptoms and objective cognitive performance. Finally, we used Venn diagrams to illustrate the overlap of objective cognitive dysfunction, subjective cognitive symptoms, and PTSD symptoms.

Statistical significance was set at *p* < .05 and all tests were two-tailed. The Bonferroni correction and the Benjamini-Hochberg adjusted false discovery rate (FDR) were used to control for multiple comparisons. No imputation was performed for missing data. Data were analyzed using IBM SPSS Statistics for Mac Version 27 (IBM Corp., Armonk, NY) and Jamovi version 2.2 (The jamovi project, 2021, https://jamovi.org).

## 3 Results

### 3.1 Patient characteristics

The mean age of the COVID-19 patients was 53.4 (SD 12.8) years and 44% were men. The HOME group was younger than the ICU, WARD, and CONTROL groups (all *p* < .001). The ICU group had less education than the WARD (*p* = .002), HOME (*p* < .001), and CONTROL (*p* < .001) groups. The CONTROL group had 50.9% men and a mean age of 54.9 (12.3) years. Demographic characteristics, comorbidities, and hospital metrics are presented in Table 1.

**Table 1:** Demographic characteristics, comorbidities, and hospital metrics in COVID-19 patients and controls.

	ICU (n = 82)	WARD (n = 53)	HOME (n = 49)	*p*^a^	All COVID-19 (N = 184)	CONTROL (n = 53)	*p*^b^
**Demographic characteristics**							
Age, mean (SD), y	57.5 (11.6)	55.6 (9.6)	44.2 (13.3)	<.001	53.4 (12.8)	54.9 (12.3)	.453
Sex, male, No./total (%)	49/82 (59.8)	19/53 (35.8)	13/49 (26.5)	<.001	81/184 (44.0)	27/53 (50.9)	.373
Education, median (IQR), y	14 (12–15)	15 (13–17)	15 (14–17)	<.001	15 (12–17)	15 (13–17)	.115
**Comorbidities** No./total (%)^c^							
CCI, median (IQR)	2 (1–3) n = 81	1 (1–2)	–	.196	–	–	–
Hypertension	45/82 (54.9)	15/53 (28.3)	8/48 (16.7)	<.001	68/183 (37.2)	11/53 (20.8)	.026
Hypercholesterolemia	26/82 (31.7)	11/53 (20.8)	4/48 (8.3)	.008	41/183 (22.4)	7/53 (13.2)	.143
Heart disease	12/82 (14.6)	3/53 (5.7)	5/48 (10.4)	.262	20/183 (10.9)	1/53 (1.9)	.042
Diabetes	19/82 (23.2)	5/53 (9.4)	2/48 (4.2)	.006	26/183 (14.2)	1/53 (1.9)	.013
Malignancy	6/82 (7.3)	3/53 (5.7)	2/48 (4.2)	.760	11/183 (6.0)	1/53 (1.9)	.229
Asthma	14/82 (17.1)	14/53 (26.4)	5/48 (10.4)	.108	33/183 (18.0)	3/53 (5.7)	.027
COPD	0/82 (0.0)	3/53 (5.7)	0/48 (0.0)	.024	3/183 (1.6)	0/53 (0.0)	.348
Kidney disease	3/82 (3.7)	0/53 (0.0)	0/48 (0.0)	.153	3/183 (1.6)	0/53 (0.0)	.348
Liver disease	0/82 (0.0)	2/53 (3.8)	2/48 (4.2)	.188	4/183 (2.2)	0/53 (0.0)	.278
**Hospital metrics**							
Delirium, No./total (%)	29/82 (35.4)	–	–	–	–	–	–
Duration of hospital stay, median (IQR), d	20 (15–26)	8 (5–11)	–	<.001		–	–
Duration of supplementary oxygen, median (IQR), d	20 (14–24) n = 78	5 (1–9)	–	<.001	–	–	–
Duration of ICU stay, median (IQR), d	10.5 (6–18)	–	–	–	–	–	–
Received IMV, No./total (%)	53/82 (64.6)	–	–	–	–	–	–
Duration of IMV, median (IQR), d^d^	7 (0–14)	–	–	–	–	–	–

Abbreviations: CCI, Charlson Comorbidity Index; COPD, Chronic Obstructive Pulmonary Disease; IMV, Invasive mechanical ventilation.

a ICU vs WARD vs HOME using ANOVA, Kruskal-Wallis test, or χ^2^ test, as appropriate.

b Complete COVID-19 group vs non-COVID controls using *t*-test, Mann-Whitney test, or χ^2^ test, as appropriate.

c Data missing from one patient in the HOME group.

d The duration of IMV in days included intubation and extubation days, and all episodes in the case of several IMV episodes.

### 3.2 Subjective cognitive symptoms

**Fig. 2:**
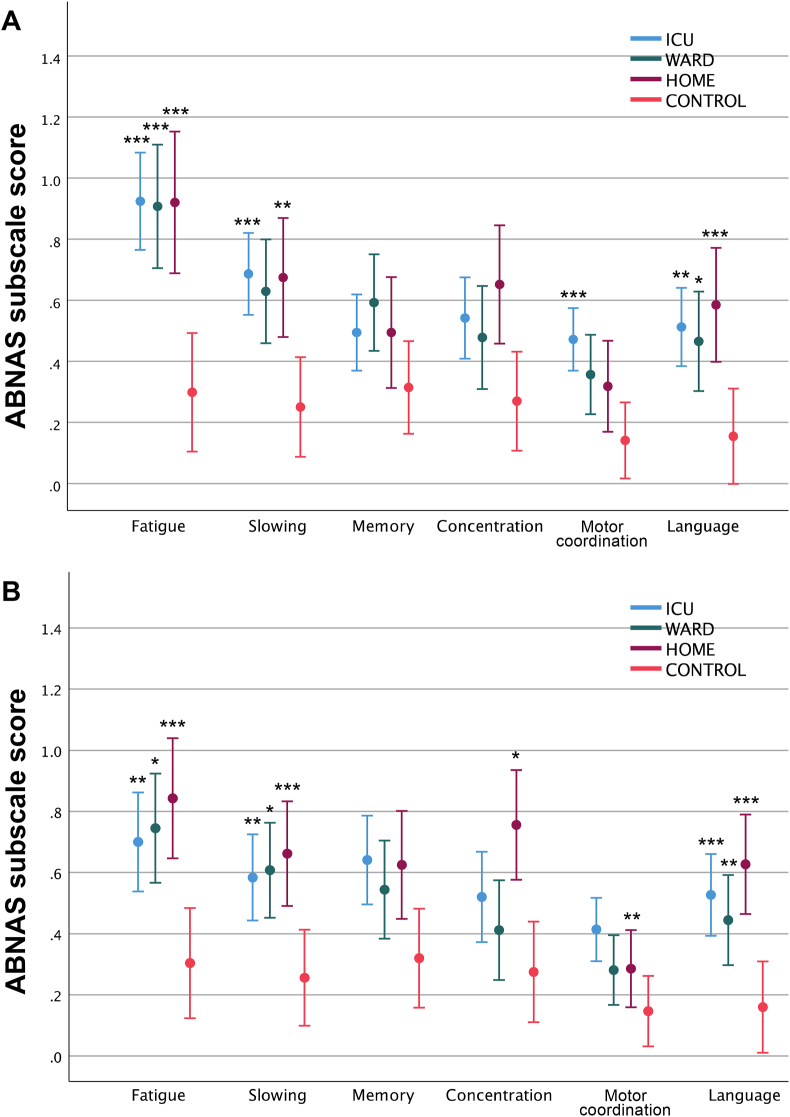
Mean ABNAS subscale scores at a) three and b) six months after COVID-19. The subscale scores were transformed to a range of 0–3 to enable comparison of subscales with differing numbers of items. Control group scores are shown in both time points. Error bars represent 95% confidence intervals. Bonferroni adjusted p values are reported. *p < .05, **p < .001, ***p < .001 compared to CONTROL. (Color: online only.). (For interpretation of the references to colour in this figure legend, the reader is referred to the Web version of this article.)

Of all COVID-19 patients, 40.6% (63/155) reported high levels of subjective cognitive symptoms (ABNAS score >15) at three months and 34.2% (53/155) at six months compared to 8.0% (4/50) of the CONTROL group. The frequencies of high ABNAS scores remained the same at three and six months (Table 2). There were no differences in ABNAS median scores between the ICU, WARD, and HOME groups (Table 2). In ABNAS subscales of fatigue (*p* < .001) and language (*p* < .001), all patient groups reported more symptoms than controls (Fig. 2). In slowing, more symptoms were reported by the ICU and HOME groups at 3 and 6 months and the WARD group at 6 months compared to controls. The ICU group reported more symptoms than controls in motor coordination at 3 and 6 months and the HOME group in concentration at 6 months.

**Table 2:** Cognitive and psychological outcomes in COVID-19 patients and controls.

	ICU (n = 82)	WARD (n = 53)	HOME (n = 49)	*p*	All COVID-19 (N = 184)	CONTROL^a^ (n = 53)	*p*
**Subjective cognitive outcome**							
ABNAS score, 3 months, median (IQR)	11 (6–23.5)	11.5 (2–27)	14 (4–23)	.845	11 (4–24)	–	–
	n = 74	n = 46	n = 35		n = 155		
ABNAS score, 6 months, median (IQR)	10.5 (5–17)	10 (3–19)	14 (5–22)	.616	11 (4–19)	4.5 (2–9)	<.001
	n = 62	n = 51	n = 42		n = 155	n = 50	
ABNAS >15, 3 months, No./total (%) [95% CI]	28/74 (37.8) [26.8–49.9]	19/46 (41.3) [27.0–56.8]	16/35 (45.7) [28.8–63.4]	.732	63/155 (40.6) [32.8–48.8]	–	–
ABNAS >15, 6 months, No./total (%) [95% CI]	20/62 (32.3) [20.9–45.3]	19/51 (37.3) [24.1–51.9]	14/42 (33.3) [19.6–49.5]	.848	53/155 (34.2) [26.8–42.2]	4/50 (8.0) [2.2–19.2]	<.001
Difference in frequency of ABNAS >15, 3 vs 6 mo, *p*	.607	.774	.070	–	–	–	–
**Objective cognitive outcome**							
t-MoCA total score, 3 months, median (IQR)	19 (17–21)	19 (17–20)	20 (19–21)	.011	19 (18–21)	–	–
	n = 77	n = 44	n = 30		n = 151		
MoCA total score, 6 months, median (IQR)	27 (25–29)	26 (25–28)	29 (27–30)	<.001	27 (25–29)	27 (25–28)	.366
	n = 72	n = 49	n = 45		n = 166	n = 48	
t-MoCA ≤19, 3 months, No./total (%) [95% CI]	41/77 (53.2) [41.5–64.7]	27/44 (61.4) [45.5–75.6]	9/30 (30.0) [14.7–49.4]	.025	77/151 (51.0) [42.7–59.2]	–	–
MoCA ≤25, 6 months, No./total (%) [95% CI]	26/72 (36.1) [25.1–48.3]	17/49 (34.7) [21.7–49.6]	4/45 (8.9) [2.5–21.2]	.003	47/166 (28.3) [21.6–35.8]	15/48 (31.3) [18.7–46.3]	.693
Difference in frequency of impaired MoCA scores, 3 vs 6 months, *p*	.007	.003	.008	–	–	–	–
**Depressive symptoms**							
Depression screen ≥1, 3 months, No./total (%) [95% CI]	29/74 (39.2) [28.0–51.2]	18/46 (39.1) [25.1–54.6]	10/35 (28.6) [14.6–46.3]	.520	57/155 (36.8) [29.2–44.9]	12/48 (25) [13.6–39.6]	.132
PHQ-9 ≥ 10, 6 months, No./total (%) [95% CI]	8/62 (12.9) [5.7–23.9]	8/51 (15.7) [7.0–28.6]	8/42 (19.0) [8.6–34.1]	.696	24/155 (15.5) [10.2–22.2]	1/50 (2.0) [0.1–10.6]	.011
**PTSD symptoms**							
IES-6 > 1.75, 3 months, No./total (%) [95% CI]	24/74 (32.4) [22.0–44.3]	13/46 (28.3) [16.0–43.5]	6/35 (17.1) [6.6–33.6]	.249	43/155 (27.7) [20.9–35.5]	–	–
IES-6 > 1.75, 6 months, No./total (%) [95% CI]	13/62 (21.0) [11.7–33.2]	10/49 (20.4) [10.2–34.3]	7/42 (16.7) [7.0–31.4]	.851	30/153 (19.6) [13.6–26.8]	2/49 (4.1) [0.5–14.0]	.010
Difference in frequency of IES-6 > 1.75, 3 vs 6 months, *p*	.109	.344	.687	–	–	–	–

Abbreviations: ABNAS, A-B Neuropsychological Assessment Schedule; MoCA, Montreal Cognitive Assessment; PHQ-9, Patient Health Questionnaire-9; IES-6, Impact of Event Scale-6.

^b^ McNemar test *p* values.

a Data collected once for the CONTROL group.

### 3.3 Objective cognitive outcome

The 3-month cognitive assessment took place on average 108 (SD 18) days after hospital discharge for ICU and 152 (41) days for WARD patients and 181 (29) days after positive COVID-19 test result for HOME patients. Cognitive impairment was observed in 77 of 151 (51.0%) patients (Table 2). A statistically significant difference in the mean t-MoCA scores emerged between ICU (19.1 [95% CI 18.5 to 19.6]), WARD (18.7 [18.0 to 19.3]), and HOME (20.2 [19.7 to 20.7]) groups (*p* = .011, η^2^ 0.06). The HOME group scores were higher than ICU (*p* = .042) and WARD (*p* = .009) groups. Age (*p* = .015, η^2^ 0.04) and education (*p* < .001, η^2^ 0.143) were significantly related to t-MoCA score. After adjusting for age and education, a significant group effect remained (*p* = .038, η^2^ 0.044) but no significant post hoc pairwise differences were found after adjusting for multiple comparisons.

The 6-month cognitive assessment occurred on average 182 (18) days after hospital discharge for ICU and 198 (17) days for WARD patients and 220 (34) days after positive COVID-19 test result for HOME patients. Cognitive impairment was observed in 47 of 166 (28.3%) patients and 15 of 48 (31.3%) controls (Table 2). The frequency of objective cognitive impairment decreased in all COVID-19 groups from 3 to 6 months (Table 2). At 6 months, there was a statistically significant difference in the mean MoCA scores between ICU (26.4 [25.7 to 27.0]), WARD (26.3 [25.7 to 26.8]), HOME (28.2 [27.6 to 28.7]), and CONTROL (26.5 [25.8 to 27.2]) groups (*p* < .001, η^2^ 0.096). In the HOME group, scores were higher than in ICU (*p* < .001), WARD (*p* < .001), and CONTROL (*p* = .003) groups. Age (*p* = .039, η^2^ 0.02) and education (*p* < .001, η^2^ 0.171) were significantly related to MoCA score. After adjusting for age and education, differences in MoCA scores were statistically significant (*p* = .006, η^2^ 0.058) with the HOME group having higher scores than the WARD (*p* = .006) and CONTROL (*p* = .024) groups.

### 3.4 Associations of subjective cognitive symptoms with psychological symptoms, demographic factors, hospital metrics, and objective cognitive impairment

IES-6 scores of >1.75 were found in 27.7% of patients at 3 months and 19.6% at 6 months (Table 2). IES-6 scores correlated with ABNAS in all COVID-19 groups at six months and in the ICU and WARD groups at three months (Table 3). On the Depression screen at three months, 36.8% of COVID-19 patients were classified as being at risk of depressive symptoms (Table 2). PHQ-9 scores of ≥10 were discovered in 15.5% of COVID-19 patients at 6 months and 2.0% of controls (Table 2). Both depression scores correlated with ABNAS scores in all COVID-19 groups (Table 3).

**Table 3:** Correlations of ABNAS scores to cognitive, psychological, demographic, and admission variables.

	ICU		WARD		HOME		CONTROL
	3 months^a^	6 months^b^	3 months^c^	6 months^d^	3 months^e^	6 months^f^	ABNAS^g^
ABNAS, 6 months	.792 *** n = 59	–	.669 ***	–	.820 *** n = 31	–	–
t-MoCA, 3 months	−.253^†^ n = 73	−.179 n = 58	−.151 n = 41	−.238 n = 43	−.014 n = 26	.034 n = 28	–
MoCA, 6 months	−.051 n = 65	.041 n = 58	−.100 n = 43	−.114 n = 47	.079 n = 32	−.106 n = 40	−.081 n = 45
Depression screen, 3 months	.519 ***	.338 * n = 59	.587 ***	.491 ** n = 46	.439 *	.263 n = 31	.411 ** n = 48
PHQ-9, 6 months	.590 *** n = 59	.816 ***	.651 ***	.838 ***	.653 *** n = 31	.825 ***	.686 ***
IES-6, 3 months	.642 ***	.540 *** n = 59	.599 ***	.388 * n = 46	.379 *	.256 n = 31	–
IES-6, 6 months	.517 *** n = 59	.653 ***	.379 * n = 45	.557 *** n = 49	.534 ** n = 31	.657 ***	.400 ** n = 49
Length of hospital stay	−.040	−.044	.017	−.192	–	–	–
Age	.097	−.015	−.053	−.032	−.003	−.248	−.216
Education	−.071	.189 n = 61	−.191 n = 45	−.190 n = 50	.162	−.213	−.158

*p < .05, **p < .001, ***p < .001. ^†^*p* = .05. FDR-adjusted p-values are reported.

a n = 74.

b n = 62.

c n = 46.

d n = 51.

e n = 35.

f n = 42.

g n = 50 unless otherwise specified.

Age and education did not correlate with ABNAS scores (Table 3). Sex was associated with ABNAS in COVID-19 patients: women (median 14 [IQR 6–27]) reported more subjective cognitive symptoms than men (median 10 [4–17.75]) at three months (*p* = .034). A statistically significant difference was detected in the ABNAS score in the ICU group (women: median 15 [7.75–34.5]; men: median 10 [4.25–17]; *p* = .018) but not in the WARD or HOME groups. At six months, there were no sex differences in ABNAS scores in patient groups or controls.

No correlations were found between the length of hospital stay and ABNAS. In ICU patients, delirium (patients with and without delirium median 10.50 [5.75–28.5] and 11.50 [5.25–19.5], respectively, *p* = .431) and IMV (with and without IMV median 12 [5–25] and 11 [7–23], respectively, *p* = .784) were not associated with ABNAS.

There was a correlation with *p* = .05 between MoCA and ABNAS at 3 months in the ICU group (Table 3). Because of strong associations between ABNAS and IES-6, additional partial correlation analyses controlling for age, education, and IES-6 were performed. In the ICU group, the correlation between ABNAS and MoCA 3-month scores was not statistically significant after controlling for age, education, and IES-6 3-month score (r = −0.222, *p* = .065). No statistically significant partial correlations between MoCA and ABNAS were found at 3 or 6 months in the ICU, WARD, or HOME groups, or in the group of all COVID-19 patients combined.

**Fig. 3:**
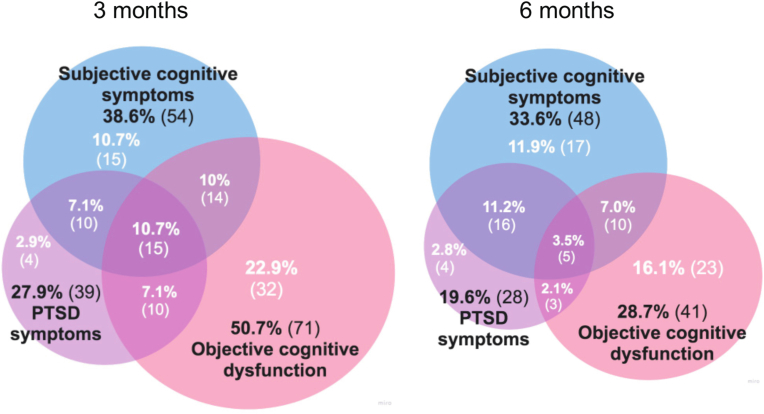
Venn diagrams of the overlap of subjective cognitive symptoms, objective cognitive dysfunction, and PTSD symptoms. Subjective cognitive symptoms were defined as an ABNAS score >15, objective cognitive dysfunction as a t-MoCA score ≤19 (3 months) and a MoCA score ≤25 (6 months), and significant PTSD symptoms as an IES-6 mean score >1.75. The 3-month (n = 140) and 6-month (n = 143) follow-ups are shown. (Color: online only.). (For interpretation of the references to colour in this figure legend, the reader is referred to the Web version of this article.)

The proportions of patients with significant subjective and objective cognitive symptoms and PTSD symptoms are described in Venn diagrams (Fig. 3).

## 4 Discussion

In this prospective single-center study of 184 COVID-19 patients and 53 controls we observed a poor congruence of subjective and objective cognitive function test results and an association of subjective cognitive impairment with depressive and PTSD symptoms in a six-month follow-up. Over one-third of patients with COVID-19, regardless of the acute disease severity, reported high levels of subjective cognitive impairment in a six-month follow-up. Objective cognitive impairment was observed in more than half of ICU and WARD patients and one-third of HOME patients at 3 months. At 6 months the cognitive performance of all three COVID-19 groups was similar (ICU and WARD) or better (HOME) than non-COVID controls. The frequency of objective but not subjective cognitive impairment decreased from 3 to 6 months. Depressive and PTSD symptoms were equally reported by all COVID-19 groups. Subjective cognitive symptoms were associated with depressive and PTSD symptoms, and in the ICU group, female sex. No associations were found between subjective cognitive impairment and objective cognitive impairment, length of hospital stay, delirium, IMV, age, or education.

The proportion of high levels of subjective cognitive symptoms in our study was approximately 30–40%, similar across the severity of acute disease, and stable in follow-up. Our findings differ from some studies where patients with more severe COVID-19 have reported more subjective symptoms (Pilotto et al., 2021; Halpin et al., 2021) but are in line with others which discovered similar rates of subjective cognitive deficits regardless of disease severity (Blackmon et al., 2022; Rass et al., 2022). Moreover, our results strengthen the finding that COVID-19 patients not requiring hospitalization can also manifest subjective cognitive symptoms (Vanderlind et al., 2021).

COVID-19 patients reported more subjective cognitive symptoms than controls especially in the domains of fatigue, slowing, and language. Fatigue has been one of the most reported symptoms after COVID-19 (Graham et al., 2021). An interesting finding was the high amount of subjective language symptoms among COVID-19 patients, which warrants further research. In the concentration domain, the HOME group reported the most symptoms, which may reflect larger demands in work and daily life as they were the youngest group. Problems in motor coordination, on the other hand, were reported by the ICU group. Muscular weakness and decreased mobility are known symptoms of post-intensive care syndrome (Needham et al., 2012). Interestingly, COVID-19 patients’ subjective symptoms in memory did not differ from non-COVID controls. Blackmon et al. (2022) reported that 30–40% of COVID-19 patients showed subjective memory and attention problems, but they had no data on controls. This highlights the importance of a control group in COVID-19 studies. During a pandemic, even healthy individuals without coronavirus disease may experience subjective difficulties relating to the overall burden of the pandemic (e.g., restrictions and social isolation) or other, unexamined factors. Given the observed psychological distress in the general population during the pandemic (Patel et al., 2022), problems in cognitive functioning are also possible.

In objective cognitive screening at three months, more than half of ICU and WARD and almost one-third of HOME patients had t-MoCA scores in the impaired range. The percentages of impaired MoCA scores decreased in follow-up. At six months, the rates of impaired cognitive performance in the ICU and WARD groups correspond to the findings of previous studies of hospitalized COVID-19 patients (Morin et al., 2021; Nersesjan et al., 2022; Raman et al., 2021). However, in our study, a similar proportion of the non-COVID controls also had MoCA scores in the impaired range. It is noteworthy that our non-COVID control sample consisted of individuals with no acute illness, whereas some studies have included patients hospitalized for other diseases as control subjects (Nersesjan et al., 2022).

Subjective cognitive symptoms were not associated with objective cognitive screening, length of hospital stay, delirium, or IMV. Some previous studies have found subjective cognitive complaints to correlate with objectively measured cognitive impairment (Miskowiak et al., 2021, 2022) whereas others (Almeria et al., 2020; Costas-Carrera et al., 2022) have not. Previous studies have reported higher incidences of either objective (Ceban et al., 2022; Mendez et al., 2022) or subjective cognitive dysfunction (Venturelli et al., 2021). Assessment methods and the definitions of impairment vary across studies. Self-reported symptoms do not necessarily manifest as impaired performance in neuropsychological tests, especially in a short cognitive screen as used in the present study. Unmeasured factors may also affect cognitive assessment (Valdes et al., 2022).

Psychological factors may also have a role in persisting subjective cognitive complaints after COVID-19 (Krishnan et al., 2022). In our study, subjective cognitive complaints were associated with depressive and PTSD symptoms, which was expected since psychological factors have been shown to associate with subjective cognitive complaints (Almeria et al., 2020; Brown et al., 2022; Henneghan et al., 2022; Liyanage-Don et al., 2022). The relationship between disease-related characteristics and subjective cognitive symptoms is still unclear. Consistent with our results, disease-related characteristics were not associated with self-reported fatigue in a previous study (Townsend et al., 2020). However, a greater number of COVID-related symptoms during the infection correlated with more subjective cognitive problems in another study (Henneghan et al., 2022). In our study, women in the ICU group reported more subjective cognitive symptoms than men. Female sex has previously been reported to be associated with incomplete recovery (Evans et al., 2021), more frequent subjective cognitive decline (Ferrucci et al., 2021; Liyanage-Don et al., 2022), and more severe fatigue after COVID-19 (Halpin et al., 2021; Townsend et al., 2020).

The strengths of our study include using a non-COVID control group which allows comparison to individuals who have been exposed to the indirect, societal factors associated with the pandemic. This is one of the first studies, to our knowledge, to investigate objective and subjective cognitive outcomes in several levels of COVID-19 severity. Additionally, the multidisciplinary approach allowed a comprehensive examination of clinical, cognitive, and psychological factors.

There are several limitations to this study. First, the home-isolated group was the youngest and predominantly female, whereas the ICU group was predominantly male with the lowest education level, reflecting the risk factors for severe COVID-19 infection. There is a rather large sex difference in the home-isolated group with a high number of female subjects. In previous research, females have reported more long-term COVID-19 symptoms than males, which may have several possible explanations such as sex-related immune system differences or gender-related social factors (Pela et al., 2022). We cannot exclude selection bias, inherent to clinical research. Second, the MoCA is a cognitive screening instrument with limited sensitivity to detect subtle cognitive deficits in younger, non-neurodegenerative populations such as our cohort (Ceban et al., 2022). In our earlier study, we found impairment especially in the ICU-treated group in domains of attention and executive functions, which are difficult to evaluate with a screening test (Ollila et al., 2022). We used both the telephone and in-person versions of the MoCA, which limits the comparability of the results. Third, there were missing data during follow-up especially for the HOME group. Fourth, we cannot exclude volunteer bias, as subjects concerned about their cognition may have been more interested in participating the study. Fifth, we did not use an antibody test to confirm that the controls had not had an asymptomatic COVID-19 infection. Thus, we cannot fully exclude the possibility of a previous asymptomatic infection in the non-COVID controls. Sixth, to our knowledge, the ABNAS was used for the first time to assess subjective cognitive functioning after COVID-19. However, it is one of the methods recommended by the NEUROCovid Neuropsychology Taskforce (Cysique et al., 2021). Finally, this is a single-center study which limits the generalizability of our results.

## 5 Conclusions

In conclusion, long-term subjective cognitive impairment and psychological symptoms were present in COVID-19 patients regardless of the severity of the acute disease. Subjective cognitive symptoms were associated with depressive and PTSD symptoms and female sex, but not with objective cognitive screening. Objective neuropsychological assessment and self-reported measures of cognition seem to assess different constructs, although providing valuable information on different aspects of functioning. It is important to thoroughly assess patients reporting long-term subjective symptoms and screen for underlying mental health related factors such as depression or PTSD.

## Funding

This work was supported by Helsinki University Hospital government funding for university level research (TYH2021310) and Nordforsk. R. Pihlaja was supported by the 10.13039/501100003125Finnish Cultural Foundation (grant number 00220808), Neurocenter, Helsinki University Hospital and the Department of Anaesthesiology, Intensive Care and Pain Medicine, Helsinki University Hospital. No other disclosures are reported. The sources of funding had no role in study design, the collection, analysis, and interpretation of data, the writing of the report, and the decision to submit the article for publication. Open access funded by Helsinki University Library.

## Declaration of competing interest

The authors declare the following financial interests/personal relationships which may be considered as potential competing interests: Riikka Pihlaja reports financial support was provided by 10.13039/501100003125Finnish Cultural Foundation. Riikka Pihlaja reports financial support was provided by 10.13039/100008376HUS Helsinki University Hospital. All authors report financial support was provided by Nordforsk. All authors report financial support was provided by Helsinki University Hospital. Johanna Hastbacka reports a relationship with Paion AG that includes: consulting or advisory.

## Data Availability

Data not publicly available, because consent for public sharing was not included in the original consent formulation. Data can be partially shared within the EU/EESC upon request for research purposes

## References

[bib1] Aldenkamp A.P., Baker G., Pieters M.S., Schoemaker H.C., Cohen A.F., Schwabe S. (1995). The Neurotoxicity Scale: the validity of a patient-based scale, assessing neurotoxicity. Epilepsy Res..

[bib2] Aldenkamp A.P., van Meel H.F., Baker G.A., Brooks J., Hendriks M.P. (2002). The A-B neuropsychological assessment schedule (ABNAS): the relationship between patient-perceived drug related cognitive impairment and results of neuropsychological tests. Seizure.

[bib3] Alhazzani W., Evans L., Alshamsi F., Moller M.H., Ostermann M., Prescott H.C., Arabi Y.M., Loeb M., Ng Gong M., Fan E., Oczkowski S., Levy M.M., Derde L., Dzierba A., Du B., Machado F., Wunsch H., Crowther M., Cecconi M., Koh Y., Burry L., Chertow D.S., Szczeklik W., Belley-Cote E., Greco M., Bala M., Zarychanski R., Kesecioglu J., McGeer A., Mermel L., Mammen M.J., Nainan Myatra S., Arrington A., Kleinpell R., Citerio G., Lewis K., Bridges E., Memish Z.A., Hammond N., Hayden F.G., Alshahrani M., Al Duhailib Z., Martin G.S., Kaplan L.J., Coopersmith C.M., Antonelli M., Rhodes A. (2021). Surviving sepsis campaign guidelines on the management of adults with coronavirus disease 2019 (COVID-19) in the ICU: first update. Crit. Care Med..

[bib4] Almeria M., Cejudo J.C., Sotoca J., Deus J., Krupinski J. (2020). Cognitive profile following COVID-19 infection: clinical predictors leading to neuropsychological impairment. Brain Behav. Immun. Health.

[bib5] Arroll B., Khin N., Kerse N. (2003). Screening for depression in primary care with two verbally asked questions: cross sectional study. BMJ.

[bib6] Becker J.H., Lin J.J., Doernberg M., Stone K., Navis A., Festa J.R., Wisnivesky J.P. (2021). Assessment of cognitive function in patients after COVID-19 infection. JAMA Netw. Open.

[bib7] Blackmon K., Day G.S., Powers H.R., Bosch W., Prabhakaran D., Woolston D., Pedraza O. (2022). Neurocognitive screening in patients following SARS-CoV-2 infection: tools for triage. Res. Sq..

[bib8] Brown L.A., Ballentine E., Zhu Y., McGinley E.L., Pezzin L., Abramoff B. (2022). The unique contribution of depression to cognitive impairment in Post-Acute Sequelae of SARS-CoV-2 infection. Brain Behav. Immun. Health.

[bib9] Ceban F., Ling S., Lui L.M.W., Lee Y., Gill H., Teopiz K.M., Rodrigues N.B., Subramaniapillai M., Di Vincenzo J.D., Cao B., Lin K., Mansur R.B., Ho R.C., Rosenblat J.D., Miskowiak K.W., Vinberg M., Maletic V., McIntyre R.S. (2022). Fatigue and cognitive impairment in Post-COVID-19 Syndrome: a systematic review and meta-analysis. Brain Behav. Immun..

[bib10] Chou S.H., Beghi E., Helbok R., Moro E., Sampson J., Altamirano V., Mainali S., Bassetti C., Suarez J.I., McNett M., Consortium G.C.-N., Consortium E. (2021). Global incidence of neurological manifestations among patients hospitalized with COVID-19-A report for the GCS-NeuroCOVID consortium and the ENERGY consortium. JAMA Netw. Open.

[bib11] Costas-Carrera A., Sanchez-Rodriguez M.M., Canizares S., Ojeda A., Martin-Villalba I., Prime-Tous M., Rodriguez-Rey M.A., Segu X., Valdesoiro-Pulido F., Borras R., Peri J.M., Vieta E. (2022). Neuropsychological functioning in post-ICU patients after severe COVID-19 infection: the role of cognitive reserve. Brain Behav. Immun. Health.

[bib12] Cysique L.A., Łojek E., Cheung T.C., Cullen B., Egbert A.R., Evans J., Garolera M., Gawron N., Gouse H., Hansen K., Holas P., Hyniewska S., Malinowska E., Marcopulos B.A., Merkley T.L., Muñoz-Moreno J.A., Ramsden C., Salas C., Sikkes S.A.M., Silva A.R., Zouhar I. (2021). Assessment of neurocognitive functions, olfaction, taste, mental, and psychosocial health in COVID-19 in adults: recommendations for harmonization of research and implications for clinical practice. J. Int. Neuropsychol. Soc..

[bib13] Daroische R., Hemminghyth M.S., Eilertsen T.H., Breitve M.H., Chwiszczuk L.J. (2021). Cognitive impairment after COVID-19-A review on objective test data. Front. Neurol..

[bib14] Davis H.E., Assaf G.S., McCorkell L., Wei H., Low R.J., Re'em Y., Redfield S., Austin J.P., Akrami A. (2021). Characterizing long COVID in an international cohort: 7 months of symptoms and their impact. EClinicalMedicine.

[bib15] Evans R.A., McAuley H., Harrison E.M., Shikotra A., Singapuri A., Sereno M., Elneima O., Docherty A.B., Lone N.I., Leavy O.C., Daines L., Baillie J.K., Brown J.S., Chalder T., De Soyza A., Diar Bakerly N., Easom N., Geddes J.R., Greening N.J., Hart N., Heaney L.G., Heller S., Howard L., Hurst J.R., Jacob J., Jenkins R.G., Jolley C., Kerr S., Kon O.M., Lewis K., Lord J.M., McCann G.P., Neubauer S., Openshaw P.J.M., Parekh D., Pfeffer P., Rahman N.M., Raman B., Richardson M., Rowland M., Semple M.G., Shah A.M., Singh S.J., Sheikh A., Thomas D., Toshner M., Chalmers J.D., Ho L.P., Horsley A., Marks M., Poinasamy K., Wain L.V., Brightling C.E., Group P.-C.C. (2021). Physical, cognitive, and mental health impacts of COVID-19 after hospitalisation (PHOSP-COVID): a UK multicentre, prospective cohort study. Lancet Respir. Med..

[bib16] Ferrucci R., Dini M., Groppo E., Rosci C., Reitano M.R., Bai F., Poletti B., Brugnera A., Silani V., D'Arminio Monforte A., Priori A. (2021). Long-lasting cognitive abnormalities after COVID-19. Brain Sci..

[bib17] Ferrucci R., Dini M., Rosci C., Capozza A., Groppo E., Reitano M.R., Allocco E., Poletti B., Brugnera A., Bai F., Monti A., Ticozzi N., Silani V., Centanni S., D'Arminio Monforte A., Tagliabue L., Priori A. (2022). One-year cognitive follow-up of COVID-19 hospitalized patients. Eur. J. Neurol..

[bib18] Graham E.L., Clark J.R., Orban Z.S., Lim P.H., Szymanski A.L., Taylor C., DiBiase R.M., Jia D.T., Balabanov R., Ho S.U., Batra A., Liotta E.M., Koralnik I.J. (2021). Persistent neurologic symptoms and cognitive dysfunction in non-hospitalized Covid-19 "long haulers". Ann. Clin. Transl. Neurol..

[bib19] Halpin S.J., McIvor C., Whyatt G., Adams A., Harvey O., McLean L., Walshaw C., Kemp S., Corrado J., Singh R., Collins T., O'Connor R.J., Sivan M. (2021). Postdischarge symptoms and rehabilitation needs in survivors of COVID-19 infection: a cross-sectional evaluation. J. Med. Virol..

[bib20] Hampshire A., Trender W., Chamberlain S.R., Jolly A.E., Grant J.E., Patrick F., Mazibuko N., Williams S.C., Barnby J.M., Hellyer P., Mehta M.A. (2021). Cognitive deficits in people who have recovered from COVID-19. EClinicalMedicine.

[bib21] Heesakkers H., van der Hoeven J.G., Corsten S., Janssen I., Ewalds E., Simons K.S., Westerhof B., Rettig T.C.D., Jacobs C., van Santen S., Slooter A.J.C., van der Woude M.C.E., van den Boogaard M., Zegers M. (2022). Clinical outcomes among patients with 1-year survival following intensive care unit treatment for COVID-19. JAMA.

[bib22] Henneghan A.M., Lewis K.A., Gill E., Kesler S.R. (2022). Cognitive impairment in non-critical, mild-to-moderate COVID-19 survivors. Front. Psychol..

[bib23] Hosey M.M., Leoutsakos J.S., Li X., Dinglas V.D., Bienvenu O.J., Parker A.M., Hopkins R.O., Needham D.M., Neufeld K.J. (2019). Screening for posttraumatic stress disorder in ARDS survivors: validation of the Impact of Event Scale-6 (IES-6). Crit. Care.

[bib24] Katz M.J., Wang C., Nester C.O., Derby C.A., Zimmerman M.E., Lipton R.B., Sliwinski M.J., Rabin L.A. (2021). T-MoCA: a valid phone screen for cognitive impairment in diverse community samples. Alzheimers Dement (Amst).

[bib25] Krishnan K., Miller A.K., Reiter K., Bonner-Jackson A. (2022). Neurocognitive profiles in patients with persisting cognitive symptoms associated with COVID-19. Arch. Clin. Neuropsychol..

[bib26] Kroenke K., Spitzer R.L., Williams J.B. (2001). The PHQ-9: validity of a brief depression severity measure. J. Gen. Intern. Med..

[bib27] Liyanage-Don N.A., Winawer M.R., Hamberger M.J., Agarwal S., Trainor A.R., Quispe K.A., Kronish I.M. (2022). Association of depression and COVID-induced PTSD with cognitive symptoms after COVID-19 illness. Gen. Hosp. Psychiatr..

[bib28] Lopez-Leon S., Wegman-Ostrosky T., Perelman C., Sepulveda R., Rebolledo P.A., Cuapio A., Villapol S. (2021). More than 50 long-term effects of COVID-19: a systematic review and meta-analysis. Sci. Rep..

[bib29] Majoka M.A., Schimming C. (2021). Effect of social determinants of health on cognition and risk of alzheimer disease and related dementias. Clin. Therapeut..

[bib30] Mendez R., Balanza-Martinez V., Luperdi S.C., Estrada I., Latorre A., Gonzalez-Jimenez P., Bouzas L., Yepez K., Ferrando A., Reyes S., Menendez R. (2022). Long-term neuropsychiatric outcomes in COVID-19 survivors: a 1-year longitudinal study. J. Intern. Med..

[bib31] Miskowiak K.W., Fugledalen L., Jespersen A.E., Sattler S.M., Podlekareva D., Rungby J., Porsberg C.M., Johnsen S. (2022). Trajectory of cognitive impairments over 1 year after COVID-19 hospitalisation: pattern, severity, and functional implications. Eur. Neuropsychopharmacol.

[bib32] Miskowiak K.W., Johnsen S., Sattler S.M., Nielsen S., Kunalan K., Rungby J., Lapperre T., Porsberg C.M. (2021). Cognitive impairments four months after COVID-19 hospital discharge: pattern, severity and association with illness variables. Eur. Neuropsychopharmacol.

[bib33] Morin L., Savale L., Pham T., Colle R., Figueiredo S., Harrois A., Gasnier M., Lecoq A.L., Meyrignac O., Noel N., Baudry E., Bellin M.F., Beurnier A., Choucha W., Corruble E., Dortet L., Hardy-Leger I., Radiguer F., Sportouch S., Verny C., Wyplosz B., Zaidan M., Becquemont L., Montani D., Monnet X. (2021). Four-month clinical status of a cohort of patients after hospitalization for COVID-19. JAMA.

[bib34] Nasreddine Z.S., Phillips N.A., Bédirian V., Charbonneau S., Whitehead V., Collin I., Cummings J.L., Chertkow H. (2005). The Montreal Cognitive Assessment, MoCA: a brief screening tool for mild cognitive impairment. J. Am. Geriatr. Soc..

[bib35] Needham D.M., Davidson J., Cohen H., Hopkins R.O., Weinert C., Wunsch H., Zawistowski C., Bemis-Dougherty A., Berney S.C., Bienvenu O.J., Brady S.L., Brodsky M.B., Denehy L., Elliott D., Flatley C., Harabin A.L., Jones C., Louis D., Meltzer W., Muldoon S.R., Palmer J.B., Perme C., Robinson M., Schmidt D.M., Scruth E., Spill G.R., Storey C.P., Render M., Votto J., Harvey M.A. (2012). Improving long-term outcomes after discharge from intensive care unit: report from a stakeholders' conference. Crit. Care Med..

[bib36] Nersesjan V., Fonsmark L., Christensen R.H.B., Amiri M., Merie C., Lebech A.M., Katzenstein T., Bang L.E., Kjaergaard J., Kondziella D., Benros M.E. (2022). Neuropsychiatric and cognitive outcomes in patients 6 Months after COVID-19 requiring hospitalization compared with matched control patients hospitalized for non-COVID-19 illness. JAMA Psychiatr..

[bib37] Ollila H., Pihlaja R., Koskinen S., Tuulio-Henriksson A., Salmela V., Tiainen M., Hokkanen L., Hastbacka J. (2022). Long-term cognitive functioning is impaired in ICU-treated COVID-19 patients: a comprehensive controlled neuropsychological study. Crit. Care.

[bib38] Patel K., Robertson E., Kwong A.S.F., Griffith G.J., Willan K., Green M.J., Di Gessa G., Huggins C.F., McElroy E., Thompson E.J., Maddock J., Niedzwiedz C.L., Henderson M., Richards M., Steptoe A., Ploubidis G.B., Moltrecht B., Booth C., Fitzsimons E., Silverwood R., Patalay P., Porteous D., Katikireddi S.V. (2022). Psychological distress before and during the COVID-19 pandemic among adults in the United Kingdom based on coordinated analyses of 11 longitudinal studies. JAMA Netw. Open.

[bib39] Pela G., Goldoni M., Solinas E., Cavalli C., Tagliaferri S., Ranzieri S., Frizzelli A., Marchi L., Mori P.A., Majori M., Aiello M., Corradi M., Chetta A. (2022). Sex-related differences in long-COVID-19 syndrome. J. Womens Health (Larchmt).

[bib40] Pendlebury S.T., Welch S.J., Cuthbertson F.C., Mariz J., Mehta Z., Rothwell P.M. (2013). Telephone assessment of cognition after transient ischemic attack and stroke: modified telephone interview of cognitive status and telephone Montreal Cognitive Assessment versus face-to-face Montreal Cognitive Assessment and neuropsychological battery. Stroke.

[bib41] Pilotto A., Cristillo V., Cotti Piccinelli S., Zoppi N., Bonzi G., Sattin D., Schiavolin S., Raggi A., Canale A., Gipponi S., Libri I., Frigerio M., Bezzi M., Leonardi M., Padovani A. (2021). Long-term neurological manifestations of COVID-19: prevalence and predictive factors. Neurol. Sci..

[bib42] Premraj L., Kannapadi N.V., Briggs J., Seal S.M., Battaglini D., Fanning J., Suen J., Robba C., Fraser J., Cho S.M. (2022). Mid and long-term neurological and neuropsychiatric manifestations of post-COVID-19 syndrome: a meta-analysis. J. Neurol. Sci..

[bib43] Raman B., Cassar M.P., Tunnicliffe E.M., Filippini N., Griffanti L., Alfaro-Almagro F., Okell T., Sheerin F., Xie C., Mahmod M., Mózes F.E., Lewandowski A.J., Ohuma E.O., Holdsworth D., Lamlum H., Woodman M.J., Krasopoulos C., Mills R., McConnell F.A.K., Wang C., Arthofer C., Lange F.J., Andersson J., Jenkinson M., Antoniades C., Channon K.M., Shanmuganathan M., Ferreira V.M., Piechnik S.K., Klenerman P., Brightling C., Talbot N.P., Petousi N., Rahman N.M., Ho L.P., Saunders K., Geddes J.R., Harrison P.J., Pattinson K., Rowland M.J., Angus B.J., Gleeson F., Pavlides M., Koychev I., Miller K.L., Mackay C., Jezzard P., Smith S.M., Neubauer S. (2021). Medium-term effects of SARS-CoV-2 infection on multiple vital organs, exercise capacity, cognition, quality of life and mental health, post-hospital discharge. EClinicalMedicine.

[bib44] Rass V., Beer R., Schiefecker A.J., Lindner A., Kofler M., Ianosi B.A., Mahlknecht P., Heim B., Peball M., Carbone F., Limmert V., Kindl P., Putnina L., Fava E., Sahanic S., Sonnweber T., Loscher W.N., Wanschitz J.V., Zamarian L., Djamshidian A., Tancevski I., Weiss G., Bellmann-Weiler R., Kiechl S., Seppi K., Loeffler-Ragg J., Pfausler B., Helbok R. (2022). Neurological outcomes 1 year after COVID-19 diagnosis: a prospective longitudinal cohort study. Eur. J. Neurol..

[bib45] Renaud-Charest O., Lui L.M.W., Eskander S., Ceban F., Ho R., Di Vincenzo J.D., Rosenblat J.D., Lee Y., Subramaniapillai M., McIntyre R.S. (2021). Onset and frequency of depression in post-COVID-19 syndrome: a systematic review. J. Psychiatr. Res..

[bib46] Schou T.M., Joca S., Wegener G., Bay-Richter C. (2021). Psychiatric and neuropsychiatric sequelae of COVID-19 - a systematic review. Brain Behav. Immun..

[bib47] Taquet M., Dercon Q., Luciano S., Geddes J.R., Husain M., Harrison P.J. (2021). Incidence, co-occurrence, and evolution of long-COVID features: a 6-month retrospective cohort study of 273,618 survivors of COVID-19. PLoS Med..

[bib48] Tavares-Junior J.W.L., de Souza A.C.C., Borges J.W.P., Oliveira D.N., Siqueira-Neto J.I., Sobreira-Neto M.A., Braga-Neto P. (2022). COVID-19 associated cognitive impairment: a systematic review. Cortex.

[bib49] Townsend L., Dyer A.H., Jones K., Dunne J., Mooney A., Gaffney F., O'Connor L., Leavy D., O'Brien K., Dowds J., Sugrue J.A., Hopkins D., Martin-Loeches I., Ni Cheallaigh C., Nadarajan P., McLaughlin A.M., Bourke N.M., Bergin C., O'Farrelly C., Bannan C., Conlon N. (2020). Persistent fatigue following SARS-CoV-2 infection is common and independent of severity of initial infection. PLoS One.

[bib50] Valdes E., Fuchs B., Morrison C., Charvet L., Lewis A., Thawani S., Balcer L., Galetta S.L., Wisniewski T., Frontera J.A. (2022). Demographic and social determinants of cognitive dysfunction following hospitalization for COVID-19. J. Neurol. Sci..

[bib51] Van Kessel S., Olde Hartman T., Lucassen P., Van Jaarsveld C. (2022). Post-acute and long-COVID-19 symptoms in patients with mild diseases: a systematic review. Fam. Pract..

[bib52] Vanderlind W.M., Rabinovitz B.B., Miao I.Y., Oberlin L.E., Bueno-Castellano C., Fridman C., Jaywant A., Kanellopoulos D. (2021). A systematic review of neuropsychological and psychiatric sequalae of COVID-19: implications for treatment. Curr. Opin. Psychiatr..

[bib53] Venturelli S., Benatti S.V., Casati M., Binda F., Zuglian G., Imeri G., Conti C., Biffi A.M., Spada M.S., Bondi E., Camera G., Severgnini R., Giammarresi A., Marinaro C., Rossini A., Bonaffini P.A., Guerra G., Bellasi A., Cesa S., Rizzi M. (2021). Surviving COVID-19 in Bergamo province: a post-acute outpatient re-evaluation. Epidemiol. Infect..

[bib54] Wahlgren C., Divanoglou A., Larsson M., Nilsson E., Östholm Balkhed Å., Niward K., Birberg Thornberg U., Lilliecreutz Gudmundsson E., Levi R. (2022). Rehabilitation needs following COVID-19: five-month post-discharge clinical follow-up of individuals with concerning self-reported symptoms. EClinicalMedicine.

[bib55] WHO (2021).

[bib56] Whooley M.A., Avins A.L., Miranda J., Browner W.S. (1997). Case-finding instruments for depression. Two questions are as good as many. J. Gen. Intern. Med..

[bib57] Zhou H., Lu S., Chen J., Wei N., Wang D., Lyu H., Shi C., Hu S. (2020). The landscape of cognitive function in recovered COVID-19 patients. J. Psychiatr. Res..

